# Social Behavior Observer Checklist: Patterns of Spontaneous Behaviors Differentiate Patients With Neurodegenerative Disease From Healthy Older Adults

**DOI:** 10.3389/fneur.2021.683162

**Published:** 2021-09-07

**Authors:** Katherine P. Rankin, Gianina Toller, Lauren Gavron, Renaud La Joie, Teresa Wu, Tal Shany-Ur, Patrick Callahan, Maggie Krassner, Joel H. Kramer, Bruce L. Miller

**Affiliations:** Department of Neurology, Memory and Aging Center, University of California, San Francisco, San Francisco, CA, United States

**Keywords:** dementia, behavior, diagnosis, measurement, interpersonal, neurodegeneration, frontotemporal lobar degeneration, Alzheimer's disease

## Abstract

Neurodegenerative disease syndromes often affect personality and interpersonal behavior in addition to cognition, but there are few structured observational measures of altered social demeanor validated for this population. We developed the Social Behavior Observer Checklist (SBOCL), a 3-min checklist tool, to facilitate identification of patterns of interpersonal behavior that are diagnostically relevant to different neurodegenerative syndromes. Research assistants without formal clinical training in dementia used the SBOCL to describe participants' behavior, including 125 healthy older adults and 357 patients diagnosed with one of five neurodegenerative disease syndromes: 135 behavioral variant frontotemporal dementia (bvFTD), 57 semantic variant primary progressive aphasia (svPPA), 51 non-fluent variant PPA (nfvPPA), 65 progressive supranuclear palsy (PSP), and 49 amyloid-positive Alzheimer's disease syndrome (AD), all of whom had concurrent 3D T1 MRI scans available for voxel-based morphometry analysis. SBOCL item interrater reliability ranged from moderate to very high, and score elevations showed syndrome-specific patterns. Subscale scores derived from a degree*frequency product of the items had excellent positive predictive value for identifying patients. Specifically, scores above 2 on the Disorganized subscale, and above 3 on the Reactive and Insensitive subscales, were not seen in any healthy controls but were found in many patients with bvFTD, svPPA, nfvPPA, PSP, and AD syndromes. Both the Disorganized and Reactive subscale scores showed significant linear relationships with frontal and temporal gray matter volume that generalized across syndromes. With these initial psychometric characteristics, the SBOCL may be a useful measure to help non-experts identify patients who are appropriate for additional specialized dementia evaluation, without adding time to patient encounters or requiring the presence of an informant.

## Introduction

Different neurodegenerative syndromes are associated with development of distinct patterns of socioemotional behavior. In some cases these are the first symptoms, occurring before classic changes in cognition (1). In Alzheimer's disease syndrome, patients have been reported to become more agitated, dependent, and sensitive to criticism, as early as the MCI phase (2–4). In behavioral variant frontotemporal dementia syndrome (bvFTD) and in right-temporal predominant cases of semantic variant of progressive primary aphasia (svPPA), patients often present with reduced socioemotional sensitivity, blunted or disorganized emotional responses, compulsiveness, and inappropriate social behavior as early features of disease (5, 6). While progressive supranuclear palsy (PSP) is considered to be a primarily motor disorder, patients with PSP may present with a frontal dysexecutive syndrome that can include symptoms of social disinhibition, apathy, and poor theory of mind, similar to bvFTD (7, 8).

Front-line non-specialist clinicians such as primary care providers typically are not equipped to recognize how these characteristic behavior patterns provide key diagnostic information (9). Particularly early in the disease course when cognitive deficits are not obvious, these patients' socioemotional symptoms can lead clinicians to misclassify them as having a psychiatric rather than neurodegenerative condition (10, 11). While several types of face-to-face neuropsychological assessment tools have been used in clinical research studies to capture patterns of socioemotional behaviors in patients with neurodegenerative syndromes (12, 13), these tools are often impractical in other settings because they require significant assessment time and expertise. In research settings, informant interviews such as the Neuropsychiatric Inventory (NPI) (14) and the Behavioral Pathology in Alzheimer's Disease Rating Scale (BEHAVE-AD) (15) are a common approach, and remain the gold standard in the field. Informant questionnaires such as the Interpersonal Reactivity Index (16), Revised Self-Monitoring Scale (RSMS) (17), Interpersonal Adjectives Scales (IAS) (18), and the Behavior Inhibition/Activation questionnaire (BIS/BAS) (19), are also used to evaluate behavior change in individuals at risk for neurodegenerative conditions (2, 20–22). However, clinical evaluations of these individuals are commonly conducted without the benefit of an informant familiar with the patient's behavior, thus a measure that relies on a clinician's direct observations of the patient during a routine clinical encounter may be necessary.

The Social Behavior Observer Checklist (SBOCL) was initially conceptualized following an earlier investigation by our group (23) that used the Interpersonal Measure of Psychopathy (IMP) (24), an observer-based behavior checklist, to show that certain spontaneous interpersonal behaviors distinguished patients with bvFTD from those with other neurodegenerative syndromes. However, the IMP was created to capture behaviors predictive of sociopathy in cognitively normal individuals, and an important conclusion of this earlier study was that a measure specifically designed to reflect the symptoms typically observed in patients with bvFTD and other neurodegenerative syndromes would likely be more effective in identifying and discriminating these conditions. The objective of the present study was to design and validate a brief observational checklist tool that would facilitate identification of patterns of interpersonal behavior that are diagnostically relevant to different neurodegenerative syndromes, and evaluate whether the tool could be used effectively by clinical examiners with no formal training or expertise in diagnosing the behavioral and interpersonal symptoms of neurodegenerative disease.

## Materials and Methods

### Development of the SBOCL

A face-valid set of behavior items was identified on the basis of (1) a comprehensive literature review of the behavioral features seen in patients with neurodegenerative disease, (2) discussions with other specialist clinicians and clinical researchers at the UCSF Memory and Aging Center with extensive experience treating patients with neurodegenerative disease syndromes, (3) the condition that items should be objectively observable by a rater over the course of a typical 30–60 min clinical interaction with the patient, and (4) the condition that items should be understandable to a rater with no formal training in clinical terminology, behavioral observation, or dementia symptomatology. An initial, slightly shorter version of the checklist was piloted with a group of 100 patients with diverse neurodegenerative conditions, and after performing user experience interviews, two descriptors and four checklist items were added. The final version of the SBOCL takes 1–3 min to complete, and contains 14 main descriptor items on which examiners are asked to identify the degree to which the patient engaged in a behavior (e.g., “Insensitive to others' embarrassment or privacy”; “not at all,” “a little bit,” “moderately,” “severely”), In addition, there are 37 checklist items referring to specific behaviors for which the frequency of occurrence could be counted (e.g., “Made an inappropriate or embarrassing joke”; “never,” “once,” “2-3xs,” “4+”). The full version of the SBOCL can be found in Figure 4, and at https://naccdata.org/data-collection/forms-documentation/ftld-3.

### Participants

We administered the SBOCL at one timepoint for 125 neurologically healthy older adults (NC) and 357 patients whom experts had diagnosed with one of five neurodegenerative disease syndromes: 135 were diagnosed with behavioral variant frontotemporal dementia (bvFTD) (6), 57 with semantic variant primary progressive aphasia (svPPA) (25), 51 with non-fluent variant primary progressive aphasia (nfvPPA) (25), 65 had progressive supranuclear palsy (PSP) (26), and 49 patients who met clinical criteria for Alzheimer's disease syndrome (AD) (27) and had a positive amyloid-PET scan (Table 1). For inclusion in the study, patients' diagnoses had been made by a multidisciplinary team of neurologists, neuropsychologists, and nurses, following thorough neurological, neuroimaging, and cognitive evaluations. Participants were recruited for the parent study from the local San Francisco Bay Area catchment area, often directly from the dementia specialty clinic associated with this research center, and non-local patients self-referred to the research center. Healthy controls were recruited *via* local media advertisements.

**Table 1:** Demographic and clinical characteristics of patient groups.

	**NC**	**bvFTD**	**svPPA**	**nfvPPA**	**PSP**	**AD**	***P*-value**
*n*	125	135	57	51	65	49	
Age	66.03 (9.29)	62.75 (8.99)^*^	65.56 (7.65)	68.07 (8.27)	70.12 (6.82)^*^	62.88 (8.71)	<0.0001
Sex, M/F	52/73	75/60	24/33	18/33	33/32	22/27	n.s.
Education	17.52 (2.07)	16.42 (3.21)^*^	16.87 (2.85)	16.05 (3.47)^*^	16.24 (3.29)	16.70 (2.41)	<0.05
MMSE^a^	29.24 (0.87)	22.13 (6.35)	23.67 (5.73)	25.92 (3.80)	25.05 (3.80)	19.92 (5.78)	<0.0001
CDR^a^	0	1.23 (0.66)	0.68 (0.44)	0.38 (0.43)	0.81 (0.59)	0.84 (0.36)	<0.0001

*Group differences were analyzed using SAS proc GLM with Tukey post-hoc tests. NC, healthy older adults; bvFTD, behavioral variant frontotemporal dementia; svPPA, semantic variant primary progressive aphasia; nfvPPA, non-fluent variant primary progressive aphasia; PSP, progressive supranuclear palsy; AD, Alzheimer's disease; MMSE, Mini-Mental State Examination; CDR^®^, Clinical Dementia Rating. Group differs from asymptomatic non-carriers at*

* *p < 0.05*.

a *Pairwise statistical comparisons only across patient groups*.

Participants underwent a 1-h neuropsychological testing session at UCSF performed by research assistants as a part of a larger study, after which the research assistants completed the SBOCL describing the participant's behavior during that session. Raters were blind to the patient's final diagnostic assignment at the time the SBOCL was completed; however, it is likely that for many patients, cognitive and motor deficits that were easily observable during the course of the evaluation revealed that they were not in the healthy control group, and thus blinding could at best be considered partial. All patients had CDR^®^ Dementia Rating System (CDR^®^) (28) and Mini-Mental State Examination (MMSE) (29) scores obtained within 90 days of SBOCL data collection. A total of 389 participants (63 NC, 123 bvFTD, 54 svPPA, 49 nfvPPA, 53 PSP, and 47 AD) had both valid SBOCL data and a structural imaging scan that was of sufficient quality and performed on a 3T scanner, and were included in the brain-behavior analyses. The average time interval between the collection of the SBOCL and MRI acquisition was 2 days. The study was approved by the Institutional Review Board at the University of California San Francisco, and all participants or their surrogates consented to participate.

### Behavioral Data Analysis

For analysis, we created presence/absence and severity scores for each of the 14 behaviors. We calculated binary summary scores based on both descriptor and checklist item scores; if either was positive then the behavior was considered to have been present. Each behavior's severity score was derived by multiplying each descriptor score (“degree”) with the maximum score across that the checklist items for that behavior (“frequency”). The severity scores of all 14 behaviors were summed to create the SBOCL Overall Severity score.

Group differences in the presence/absence of each behavior were analyzed with Chi-square tests, and severity scores for each behavior and the Overall Severity score were analyzed with general linear models (SAS Proc GLM), controlling for age and sex. To determine if the 14 heterogeneous behaviors grouped into meaningful subscale scores according to a data-driven approach, we performed a cluster analysis across all patients using the descriptor severity scores (SAS proc VARCLUS). Because of the disproportionately larger number of patients in the bvFTD group (*n* = 135) compared to the other patient groups (*N*'s ranging from 49 to 65), we tested the model for bias by conducting a second cluster analysis across all patient groups while including only 60 randomly selected patients from the bvFTD sample, thereby better matching subgroup sizes. Cluster severity scores (degree*frequency product, averaged across behaviors belonging to that cluster) were calculated, and group differences were analyzed using GLMs. Logistic regression analysis (SAS proc LOGISTIC) and receiver operating characteristic (ROC) curves were used to determine how well each cluster subscale score distinguished each patient group from NCs.

### Interrater Reliability

During test development, a subset of 15 patients were consented to have their neuropsychological testing session video recorded, including 5 consecutively enrolled patients with bvFTD, 5 with svPPA, and 5 with AD. Two research assistants watched each video and independently rated the patient's behavior using the SBOCL. The agreement of the two video raters was determined by calculating Kendall's W (coefficient of concordance for ordinal data) for each of the descriptor items (SAS macro: %magree.sas). Then, with the expectation that the behaviors observable on the video would likely yield somewhat lower concordance when compared with the behavior experienced in the room, Kendall's W was again calculated across the ratings of the examiner in the room and the two video raters.

### Neuroimaging

#### Image Acquisition and Pre-processing

All structural T1-weighted images were acquired on 3T-scanners (Siemens Trio and Prisma) at the University of California, San Francisco. T1-weighted 3D magnetization prepared rapid gradient echo (MPRAGE) sequence was used to obtain the structural images, with acquisition parameters as follows: 160 sagittal slices, 1-mm thick, skip = 0 mm; repetition time = 2,300 ms; echo time = 2.98 ms; flip angle = 9°; field of view = 240 × 256 mm^2^; voxel size = 1 mm^3^; matrix size = 256 × 256.

The images were preprocessed using SPM12. The images were visually inspected for artifacts, and underwent bias-correction, segmentation into tissue compartments, and spatial normalization using a single generative model with the standard SPM12 parameters. The default tissue probability maps for gray matter, white matter, cerebrospinal fluid, and all other voxels from SPM12 (TPM.nii) were used. To optimize intersubject registration, each participant's image was warped to a template derived from 300 confirmed neurologically healthy older adults (ages 44–86, M ± SD: 67.2 ± 7.3; 114 males, 186 females), using affine and non-linear transformations with the help of the diffeomorphic anatomical registration through exponentiated lie algebra (DARTEL) (30) method, with standard implementation in SPM12. In all preprocessing steps, default parameters of the SPM12 toolbox were used. Total volume of each tissue compartment was calculated by applying the modulated, warped, and segmented masks for gray matter, white matter, and CSF to the corresponding probability map for that individual, and the total intracranial volume (TIV) was derived by summing the three volumes. The spatially normalized, segmented, and modulated gray matter images were smoothed with an 8-mm FWHM isotropic Gaussian kernel.

#### Image Analysis

Using the smoothed gray matter images we performed whole-brain voxel-based morphometry (VBM) analysis to investigate the gray matter correlates of each cluster. Specifically, GLMs were calculated to examine whether each subscale score predicted gray matter volume, with age, sex, and total intracranial volume (TIV) included as covariates of no interest (Main Effects Analysis). As an error check, to determine whether our brain-behavior relationships were generalizable across patient syndromes, we performed a second set of analyses and added each diagnostic group as a binary variable to the models (Diagnostic Confound Analysis). A 1000-permutation analysis was used to identify the study-specific *t*-threshold at *p* < 0.05 to correct for family-wise error (FWE) (31, 32).

## Results

### Demographic and Clinical Characteristics

Mean age was significantly younger in patients with bvFTD (M ± SD: 62.75 ± 8.99; *p* < 0.05) and significantly older in patients with PSP (70.12 ± 6.82; *p* < 0.05) compared to the NCs (66.03 ± 9.29) (Table 1), though groups did not differ by biological sex. Patients with bvFTD (16.42 ± 3.21; *p* < 0.05) and nfvPPA (16.05 ± 3.47; *p* < 0.05) had fewer years of education than the NC group (17.52 ± 2.07), though the mean difference was only one school year and thus unlikely to reflect clinically meaningful differences in this highly educated sample. Both proxies for disease severity (CDR^®^ Global score and MMSE Total score) significantly differed among patient groups, with worse scores in patients with AD and bvFTD compared to the other groups. Age, sex, and MMSE score were included as covariates of no interest in all statistical analyses.

### Group Differences in SBOCL Overall, Item, and Subscale Scores

Patients with bvFTD (45.12 ± 1.77; *p* < 0.001), svPPA (37.75 ± 2.57; *p* < 0.001), and PSP (32.65 ± 2.45) had significantly higher SBOCL overall severity score than the NC group (22.18 ± 1.93; *p* = 0.004) (Table 2). The other patient groups did not significantly differ from NCs. The majority of items showed elevated degree*frequency (i.e., severity) scores and were significantly more likely to be present in patients compared to NCs (Table 2). The items that were the least useful were “Preoccupied with time” and “Insensitive to others' embarrassment,” both of which were more severe in bvFTDs relative to controls, but were comparatively infrequent and of milder severity than the other behaviors.

**Table 2:** Overall, cluster, and item presence and severity scores for each diagnostic group.

	**NC**	**bvFTD**	**svPPA**	**nfvPPA**	**PSP**	**AD**	**Omnibus *P*-value**
*N*	125	135	57	51	65	49	
**Severity, M (SD)**							
**Overall SBOCL Severity Score**	15.61 (1.84)	48.66 (1.79)^***^	39.09 (2.71)^***^	27.64 (2.89)^*^	32.26 (2.59)^***^	36.81 (2.94)^***^	<0.0001
**Cluster 1: Disorganized score**	1.07 (0.21)	4.65 (0.21)^***^	2.80 (0.31)^***^	2.26 (0.33)^*^	3.11 (0.30)^***^	3.16 (0.34)^***^	<0.0001
Failed to adapt to structure	1.04 (0.30)	5.01 (0.29)^***^	3.25 (0.44)^**^	1.84 (0.47)	2.11 (0.42)	3.01 (0.47)^*^	<0.0001
Stimulus-bound	1.17 (0.33)	5.14 (0.32)^***^	3.43 (0.48)^**^	2.16 (0.51)	3.26 (0.46)^**^	2.68 (0.52)	<0.0001
Perseverative	1.04 (0.33)	4.63 (0.32)^***^	2.94 (0.49)^*^	1.93 (0.52)	3.03 (0.47)^**^	3.07(0.53)^*^	<0.0001
Decreased initiation	0.98 (0.37)	4.54 (0.36)^***^	2.49 (0.55)	2.92 (0.58)	4.03 (0.52)^***^	3.88 (0.59)^**^	<0.0001
Fluctuating cognition	1.06 (0.31)	4.40 (0.31)^***^	2.65 (0.46)	2.40 (0.50)	2.99 (0.44)^*^	4.46 (0.50)^***^	<0.0001
Diminished social engagement	1.16 (0.28)	4.17 (0.27)^***^	2.07 (0.42)	2.32 (0.44)	3.23 (0.40)^**^	1.87 (0.45)	<0.0001
**Cluster 2: Reactive score**	1.17 (0.15)	2.50 (0.15)^***^	3.34 (0.23)^***^	2.05 (0.25)^*^	1.99 (0.22)^*^	2.72 (0.25)^***^	<0.0001
Overly self-conscious	1.59 (0.34)	3.36 (0.33)^**^	6.35 (0.50)^***^	4.23 (0.53)^**^	3.16 (0.47)	5.27 (0.54)^***^	<0.0001
Anxious	1.14 (0.22)	1.79 (0.22)	3.17 (0.33)^***^	1.92 (0.35)	1.35 (0.31)	2.77 (0.35)^**^	<0.0001
Overly dependent	1.16 (0.25)	2.85 (0.24)^***^	3.36 (0.36)^***^	1.87 (0.39)	2.31 (0.35)	2.43 (0.39)	<0.0001
Exaggerated emotionality	0.97 (0.24)	2.87 (0.24)^***^	2.22 (0.36)	1.27 (0.38)	2.10 (0.34)	1.94 (0.39)	<0.0001
Preoccupied with time	0.99 (0.12)	1.60 (0.12)^*^	1.58 (0.18)	0.98 (0.20)	1.00 (0.18)	1.17 (0.20)	0.002
**Cluster 3: Insensitive score**	1.10 (0.14)	2.77 (0.14)^**^	1.86 (0.21)^*^	1.27 (0.22)	1.23 (0.20)	1.42 (0.23)	<0.0001
Too little self-consciousness	1.17 (0.25)	3.64 (0.25)^***^	2.75 (0.38)^*^	1.62 (0.40)	1.44 (0.36)	1.73 (0.41)	<0.0001
Insensitive to embarrassment	1.09 (0.12)	1.72 (0.12)^**^	1.41 (0.18)	1.07 (0.19)	1.09 (0.17)	1.16 (0.19)	0.0016
Overly disclosing/familiar	1.05 (0.20)	2.95 (0.20)^***^	1.42 (0.30)	1.10 (0.32)	1.17 (0.29)	1.37 (0.33)	<0.0001
**Presence**, ***N*****(%)**							
**Cluster 1: Disorganized score**	6 (5%)	105 (78%)	37 (65%)	22 (43%)	39 (60%)	28 (57%)	<0.0001
Failed to adapt to structure	1 (1%)	55 (41%)	17 (30%)	8 (16%)	10 (15%)	13 (27%)	<0.0001
Stimulus-bound	3 (2%)	61 (45%)	17 (30%)	6 (12%)	16 (25%)	8 (16%)	<0.0001
Perseverative	1 (1%)	46 (34%)	13 (23%)	6 (12%)	16 (25%)	8 (16%)	<0.0001
Decreased initiation	0 (0%)	45 (33%)	10 (18%)	11 (22%)	17 (26%)	15 (31%)	<0.0001
Fluctuating cognition	1 (1%)	47 (35%)	13 (23%)	9 (18%)	12 (19%)	18 (37%)	<0.0001
Diminished social engagement	0 (0%)	42 (31%)	5 (9%)	4 (8%)	8 (12%)	5 (12%)	<0.0001
**Cluster 2: Reactive score**	17 (14%)	49 (36%)	42 (74%)	24 (47%)	21 (32%)	25 (51%)	n.s.
Overly self-conscious	12 (10%)	32 (24%)	34 (60%)	19 (37%)	17 (26%)	22 (45%)	<0.0001
Anxious	1 (1%)	8 (6%)	11 (19%)	4 (8%)	1 (2%)	7 (14%)	<0.0001
Overly dependent	6 (5%)	26 (19%)	18 (32%)	9 (18%)	11 (17%)	9 (19%)	0.0004
Exaggerated emotionality	0 (0%)	17 (13%)	5 (9%)	1 (2%)	3 (5%)	3 (6%)	0.0009
Preoccupied with time	0 (0%)	6 (4%)	4 (7%)	0 (0%)	1 (2%)	1 (2%)	0.0389
**Cluster 3: Insensitive score**	5 (4%)	51 (38%)	13 (23%)	5 (10%)	4 (6%)	7 (14%)	<0.05
Too little self-consciousness	3 (2%)	40 (30%)	12 (21%)	5 (10%)	4 (6%)	7 (14%)	<0.0001
Insensitive to embarrassment	1 (1%)	11 (8%)	3 (5%)	1 (2%)	0 (0%)	0 (0%)	0.0042
Overly disclosing/familiar	1 (1%)	22 (16%)	5 (9%)	0 (0%)	0 (0%)	1 (2%)	<0.0001

For severity scores (i.e., the degree*frequency product score for each behavior), Dunnett-Hsu post-hoc tests were used to compare mean least-square scores between each patient group and the control group, controlling for age and sex. Cluster severity scores (Disorganized, Reactive, Insensitive) were derived from cluster modeling across all descriptors. Presence scores represent the percentage of individuals in the diagnostic group for whom the symptom was considered “present” by the examiner. NC, healthy older controls; bvFTD, behavioral variant frontotemporal dementia; svPPA, semantic variant primary progressive aphasia; nfvPPA, non-fluent variant primary progressive aphasia; PSP, progressive supranuclear palsy; AD, Alzheimer's disease; SBOCL, Social Behavior Observer Checklist. Group differs from NC at

* *p < 0.01*,

** *p < 0.001*,

*** *p < 0.0001*.

**Figure 1:**
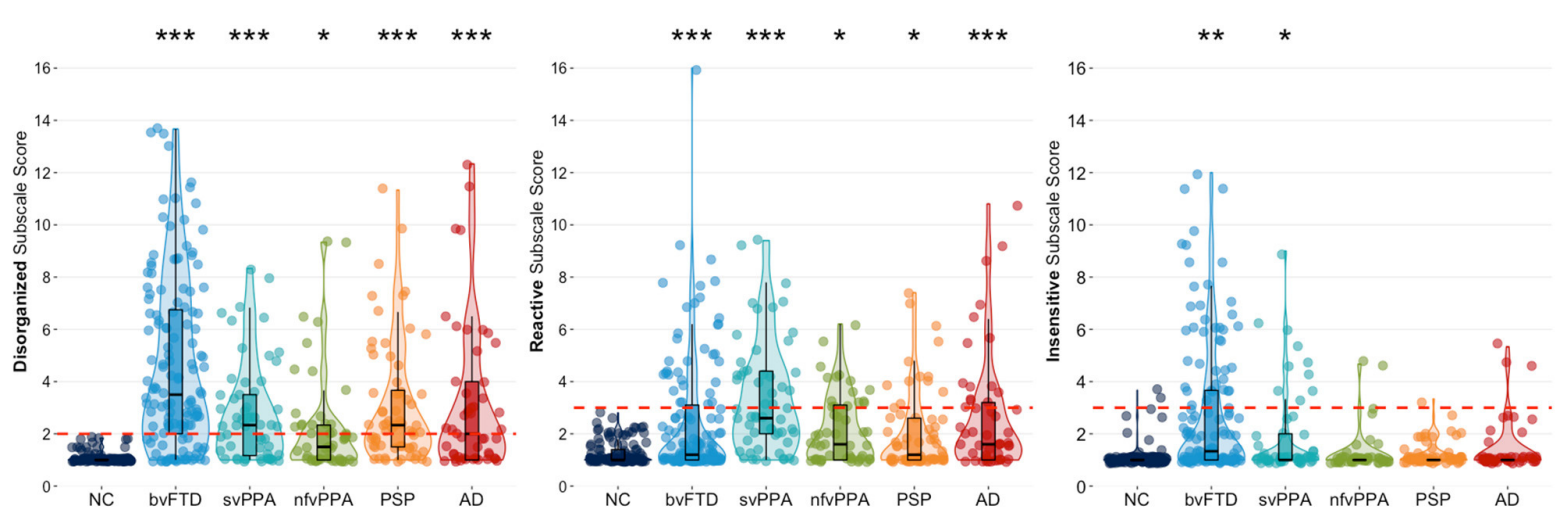
Violin boxplots of each cluster's mean severity score classified by diagnostic group. Dunnett-Hsu *post-hoc* tests were used to compare least-square mean subscale scores between each patient group and the NC group. The red dotted lines represent the proposed scoring threshold for each subscale score. NC, healthy older controls; bvFTD, behavioral variant frontotemporal dementia; svPPA, semantic variant primary progressive aphasia; nfvPPA, non-fluent variant primary progressive aphasia; PSP, progressive supranuclear palsy; AD, Alzheimer's disease. *Patient group differs from NC at *p* < 0.05; **Patient group differs from NC at *p* < 0.001; ***Patient group differs from NC at *p* < 0.0001.

Cluster modeling across all descriptors revealed that the most robust solution divided into three behavioral symptom categories: (1) **Disorganized** (Failed to adapt to structure, Stimulus-bound, Perseverative, Decreased initiation, Fluctuating level of cognitive ability, Diminished social engagement), (2) **Reactive** (Overly self-conscious, Anxious, Overly dependent, Exaggerated emotional reactivity, Preoccupied with time), and (3) **Insensitive** (Too little self-consciousness, Insensitive to others' embarrassment, Overly disclosing/inappropriately familiar). The same three cluster solution was revealed for both the full patient sample, and for analyses including only a subset of bvFTD patients to correct for any bias from the larger size of that subgroup. Our results also showed that behaviors of the Disorganized cluster occurred most frequently in patients with bvFTD (78%), followed by patients with svPPA (65%) and PSP (60%) (Table 2). In addition, we found that Disorganized symptoms were significantly more severe in patients with bvFTD (4.11 ± 0.19, *p* < 0.001) and PSP (3.14 ± 0.27, *p* < 0.05) compared to the NC group (2.02 ± 0.21) (Figure 1). The frequency of Reactive behaviors was highest in patients with svPPA (74%), AD (50%), and nfvPPA (47%). However, the severity of Reactive behaviors was significantly increased in all patient groups compared to NC. Similar to the Disorganized cluster, the number of patients showing Insensitive behaviors was highest in the bvFTD (38%) and svPPA (23%) groups; however, only patients with bvFTD (2.66 ± 0.14, *p* < 0.001) showed significantly more severe Insensitive behaviors than the NC group (1.29 ± 0.16, *p* < 0.001).

### Characteristics of Each Subscale and Descriptor for Differential Diagnosis

**Figure 2:**
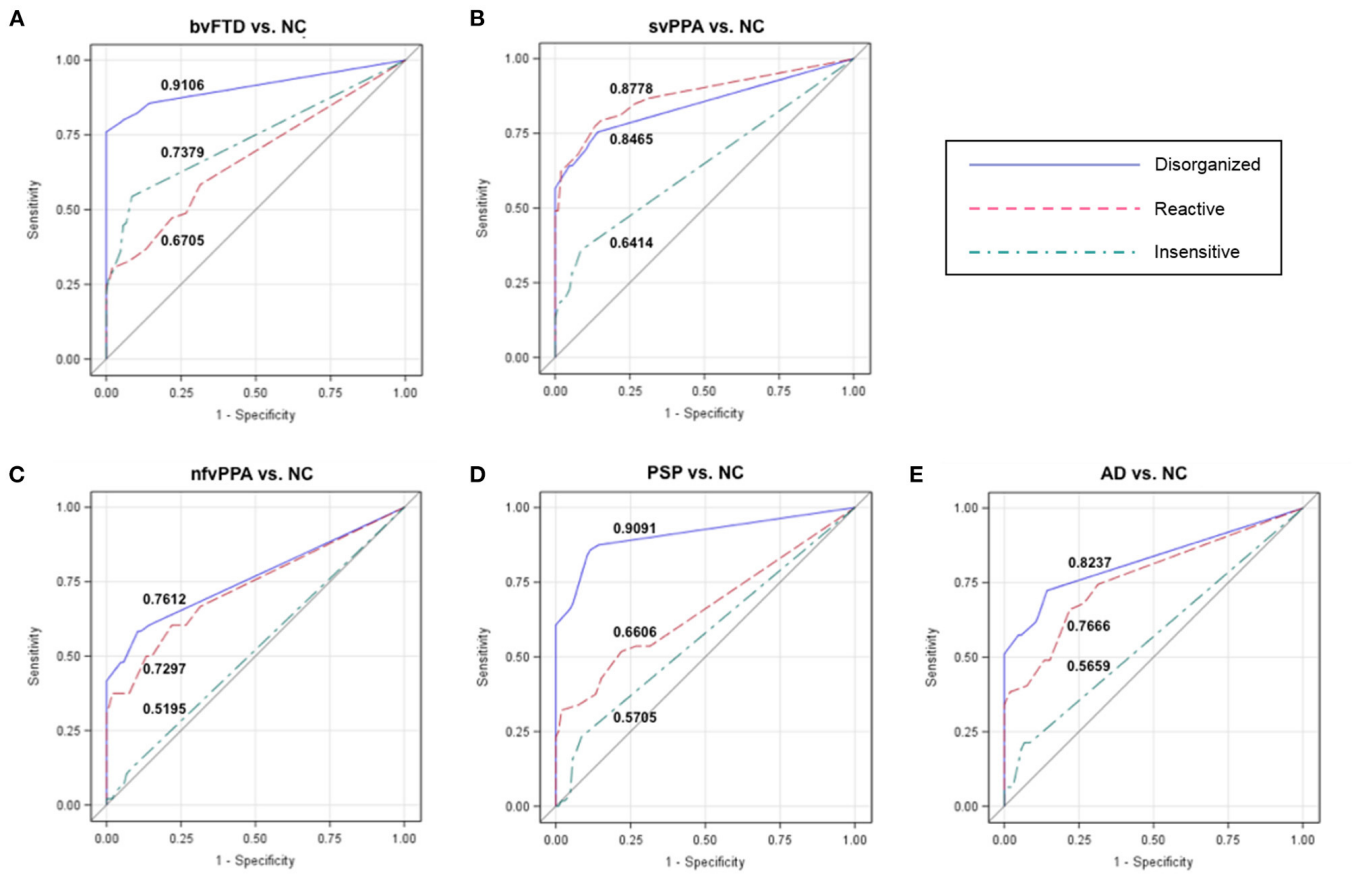
Receiver operating characteristic (ROC) curves for each patient group vs. NCs. The numbers in each graph show the ROC area under the curve (AUC) for each subscale and group comparison (patients vs. controls). The Disorganized subscale can distinguish **(A)** patients with behavioral variant frontotemporal dementia (bvFTD) from healthy controls (NC) with a probability of 91.06%, and **(D)** patients with progressive supranuclear palsy (PSP) from NC with a probability of 90.91%. The Reactive and Disorganized subscales have similar probabilities to differentiate patients with **(B)** semantic variant primary progressive aphasia (svPPA) from NC, as well as patients with **(E)** Alzheimer's disease (AD) and **(C)** non-fluent variant primary progressive aphasia (nfvPPA) from NC. NC, healthy older controls; bvFTD, behavioral variant frontotemporal dementia; svPPA, semantic variant primary progressive aphasia; nfvPPA, non-fluent variant primary progressive aphasia; PSP, progressive supranuclear palsy; AD, Alzheimer's disease.

For each cluster, we determined a cutoff score that had optimal characteristics for differential diagnosis between patients and NC. ROC analysis revealed that at a cutoff score of 2 the Disorganized subscale showed high accuracy (AUC: 91.06%) to differentiate patients with bvFTD and NC (Table 3; Figure 2A). In addition, 100% of individuals who had an abnormal score of 2 or above had a bvFTD diagnosis (positive predictive value, PPV), whereas only 15.6% of individuals who had a normal score (below 2) had a bvFTD diagnosis (false omission rate, FOR). At this threshold of 2, the subscale also showed high probability to correctly distinguish patients with PSP from NC (AUC: 90.91%; Figure 2D). The svPPA, nfvPPA, and AD groups also had a PPV of 100% and small omission rates <20%. For the Reactive cluster, we found that using a cutoff score of 3 enabled us to correctly differentiate patients with svPPA from NC in 87.78% of cases (Figure 2B), with a PPV of 100% and a FOR of 18.83%. The PPVs for the other patient groups was also 100%, but the FORs were high particularly in patients with bvFTD (50%) but also in patients with PSP (29.38%). The highest probability (AUC: 73.79%) to differentiate patients with bvFTD and NC on the Insensitive subscale was achieved using a cutoff score of 3 (Figure 2A), which was also associated with a high PPV of 93.33% but also a high FOR (43.26%). In the other patient groups, the majority of the validity scores were close to chance level (Table 3; Figures 2C,E).

**Table 3:** Subscale characteristics for each patient group vs. healthy older adults.

	**bvFTD**	**svPPA**	**nfvPPA**	**PSP**	**AD**
**Disorganized subscale (cutoff score = 2)**					
ROC AUC (%)	**91.06**	84.65	76.12	**90.91**	82.37
Positive predictive value (%)	100.00	100.00	100.00	89.58	100.00
False omission rate (%)	20.89	14.28	19.35	15.49	16.11
**Reactive subscale (cutoff score = 3)**					
ROC AUC (%)	67.05	**87.78**	72.97	66.06	76.66
Positive predictive value (%)	100.00	100.00	100.00	100.00	100.00
False omission rate (%)	50.00	18.83	21.88	29.38	20.38
**Insensitive subscale (cutoff score = 3)**					
ROC AUC (%)	**73.79**	64.14	51.95	57.05	56.59
Positive predictive value (%)	93.33	76.92	50.00	25.00	50.00
False omission rate (%)	43.26	27.81	28.24	34.41	27.38

*ROC, Receiver Operating Characteristic; AUC, Area Under the Curve; bvFTD, behavioral variant frontotemporal dementia; svPPA, semantic variant primary progressive aphasia; nfvPPA, non-fluent variant primary progressive aphasia; PSP, progressive supranuclear palsy; AD, Alzheimer's disease. Bold text indicates the diagnostic group(s) with highest relative AUC for that subscale*.

To obtain more practical information for differential diagnosis, we investigated which checklist items best discriminated each patient group from NC. The items “Failed to adapt to structure of testing” (AUC: 75.61%) and “Stimulus-bound” (AUC: 75.37%) most accurately distinguished patients with bvFTD from NC. The behaviors “Overly self-conscious” (AUC: 78.09%), “Failed to adapt to structure of testing” (AUC: 70.72%), and “Overly dependent” (AUC: 70.41%) were most sensitive to differentiate patients with svPPA and NC. Patients with nfvPPA who were described as “Overly self-conscious” (AUC: 70.67%) best differentiated from NC. Two checklist items distinguished patients with AD from NC (AUC “Self-conscious”: 73.51%; AUC “Fluctuating levels of cognitive ability”: 76.98%). Checklist items were fairly poor at differentiating patients with PSP from NCs, with AUCs of 67% and below.

### Interrater Reliability

Between the two independent video raters, Kendall's W coefficient of concordance for ordinal response for each of the SBOCL descriptor items was “very good” (0.8–1.0) or “good” (0.6–0.8) for 10 of 14 items, “moderate” (0.4–0.6) for three items, and “fair” (0.2–0.4) for one item (“Stimulus-bound behavior”) (see Table 4). When these video ratings were compared to the rating performed by the examiner who had tested the patient, the degree of concordance remained very high, with the majority of descriptor items showing “good” or “very good” concordance among the 3 raters.

**Table 4:** Interrater reliability of SBOCL descriptor items rated by non-specialist observers.

	**Video only (2 Raters)**			**Video and in room (3 Raters)**		
	**Kendall's W**	**Interpretation**	***p*-value**	**Kendall's W**	**Interpretation**	***p*-value**
Overly dependent	0.99	Very good	<0.0001	0.87	Very good	<0.0001
Fails to adapt to structure	0.95	Very good	<0.0001	0.84	Very good	<0.0001
Decreased initiation	0.90	Very good	0.0002	0.87	Very good	<0.0001
Diminished social engagement	0.88	Very good	0.0006	–		
Too little self-consciousness	0.83	Very good	0.0037	0.63	Good	0.0032
Overly self-conscious	0.79	Good	0.0117	0.81	Very good	<0.0001
Exaggerated emotional reactivity	0.78	Good	0.0158	–		
Perseverative	0.77	Good	0.0200	0.65	Good	0.0022
Fluctuating cognition	0.75	Good	0.0263	0.73	Good	0.0001
Insensitive to other's embarrassment	0.73	Good	0.0421	0.70	Good	0.0003
Anxious	0.52	Moderate	0.4316	0.48	Moderate	0.0896
Overly disclosing/familiar	0.50	Moderate	0.5000	0.46	Moderate	0.1228
Preoccupied with time	0.46	Moderate	0.5999	0.39	Fair	0.2874
Stimulus-bound	0.40	Fair	0.7538	0.37	Fair	0.3660

*During test development, a subset of 15 patients were consented to have their neuropsychological testing session video recorded, including 5 consecutively enrolled patients with bvFTD, 5 with svPPA, and 5 with AD. Two research assistants watched each video and independently rated the patient's behavior using the SBOCL. The agreement of the two video raters with each other and with the rating made by the in-room examiner was determined by calculating Kendall's W (coefficient of concordance for ordinal data) for each of the descriptor items (SAS macro: %magree.sas)*.

### Gray Matter Correlates of Each Cluster

**Figure 3:**
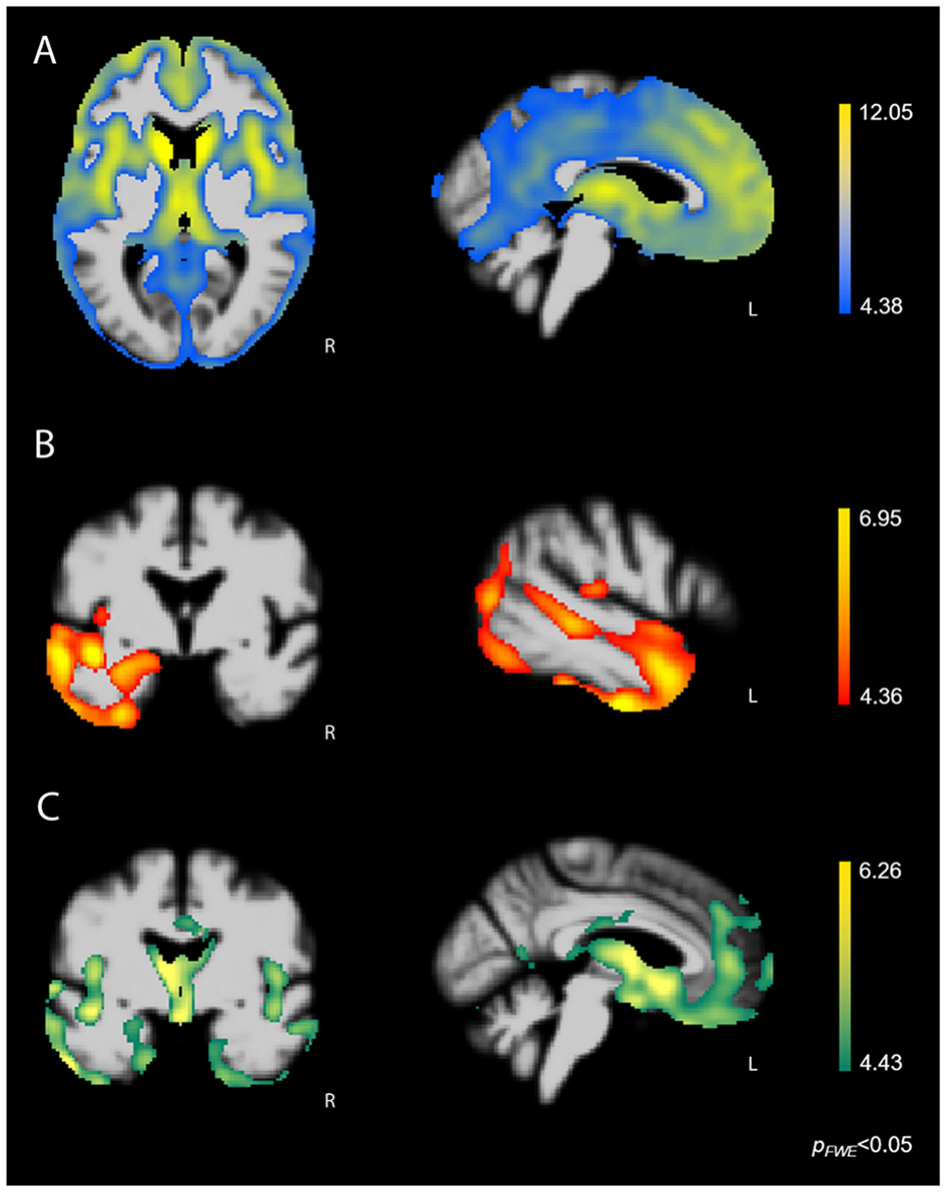
FWE-corrected *t*-maps showing regions where atrophy predicted SBOCL subscale scores. **(A)** Higher score on the Disorganized subscale corresponded with lower volume predominantly in bilateral fronto-subcortical regions, including the caudate, thalamus, anterior insula, as well as medial, lateral, and orbitofrontal cortex. **(B)** Higher score on the Reactive subscale corresponded with gray matter atrophy in the left lateral and mesial temporal lobe. **(C)** Higher score on the Insensitive subscale corresponded with volume loss in bilateral fronto-temporal and subcortical regions, including the caudate, thalamus, anterior insula, subgenual ACC, OFC, as well as medial and lateral temporal lobe. Age, sex, and TIV were included as covariates of no interest in each analysis. L, Left.

Whole-brain VBM analysis showed that higher score on the Disorganized subscale corresponded with greater atrophy in bilateral fronto-subcortical structures overlapping with the salience (SN) (33), semantic-appraisal (SAN) (34), and fronto-parietal (FPN) (35) networks. Regions included the bilateral caudate, thalamus, anterior insula, inferior and middle frontal gyri, medial and lateral superior frontal gyrus, orbitofrontal cortex (OFC), nucleus accumbens, and anterior cingulate cortex (ACC). All regions survived the FWE-corrected critical *t*-threshold (Figure 3A). These regions remained statistically significant when we added the binarized diagnostic groups (k-1) as additional confounds to the analysis. By contrast, higher score on the Reactive subscale was associated with lower volume predominantly in the left temporal lobe, including the left inferior, middle, and superior temporal gyri, temporal pole, fusiform gyrus, posterior insula, and mesial temporal lobe (amygdala, hippocampus) (Figure 3B). In addition, our results showed that higher score on the Reactive subscale corresponded with smaller volume in the left inferior and middle occipital gyri, as well as a very small cluster in the right temporal pole (all *p* < 0.05, FWE-corrected). The correspondence of the Reactive subscale with volume in the left temporal lobe suggested that this relationship may have resulted from the left temporal lobe damage seen primarily in the svPPA group (Figure 3C). Thus, we performed a hypothesis-driven error check for generalizability by including a binarized variable (svPPA [1] vs. other groups [0]) in the model. This analysis confirmed the relationship between lower score on the Reactive subscale and atrophy in the left temporal lobe, demonstrating that sufficient left temporal atrophy was found in the non-svPPA patient groups to reflect this same linear relationship. Thus, the relationship can be considered generalizable. Finally, higher score on the Insensitive subscale corresponded with volume loss in bilateral fronto-temporal and subcortical regions, including the bilateral thalamus, anterior and posterior insula, fusiform gyrus, inferior, middle, and superior temporal gyri, temporal pole, subcallosal area, caudate, mesial temporal lobe (amygdala, hippocampus, parahippocampal gyrus), OFC, and ACC (all *p* < 0.05, FWE-corrected). This atrophy pattern included many core regions of the SN and SAN that are affected in bvFTD, suggesting that these brain-behavior relationships may have been caused predominantly by the bvFTD group. Thus, we performed a error check by adding the binarized variable “bvFTD (1) vs. other groups (0)” in the analysis. Except for some small clusters in the left temporal lobe, all fronto-subcortical regions lost significance in this analysis, showing that the significant brain-behavior relationship did not generalize beyond the bvFTD group.

**Figure 4:**
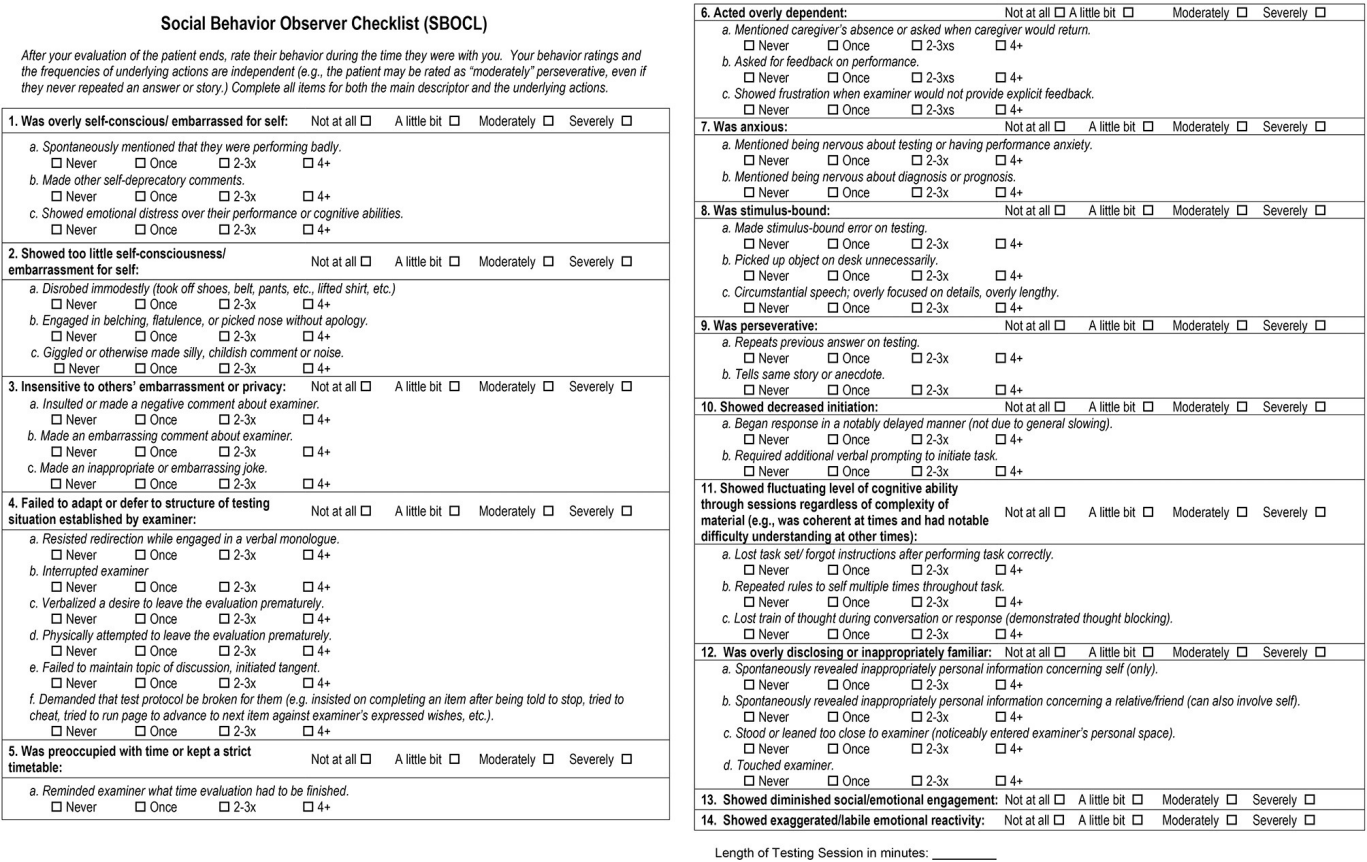
Social behavior observer checklist.

## Discussion

This study showed that neurodegenerative syndromes create distinct patterns of spontaneous social behavior symptoms that can be objectively recognized during a time-limited clinical evaluation, even by individuals without formal clinical training. The SBOCL measure, consisting of both descriptor ratings and behavior checklist items, showed strong initial interrater reliability as measured in a subset of the sample. Subscale scores derived from a degree*frequency product of the items had excellent positive predictive value for identifying patients; specifically, scores above 2 on the Disorganized subscale, and above 3 on the Reactive and Insensitive subscales, were not seen in any healthy controls but were found in large numbers of patients with bvFTD, svPPA, nfvPPA, PSP, and AD syndromes. Both the Disorganized and Reactive subscale scores showed significant linear relationships with frontal and temporal gray matter volume that generalized across diagnostic syndromes. With these psychometric characteristics, the SBOCL may help non-experts recognize and appropriately refer patients for specialized dementia evaluation even when cognitive test results are unavailable.

The SBOCL measure was designed to assist individuals without specialized medical training in the clinical differential diagnosis of dementia syndromes to identify spontaneous behavior symptoms that may be diagnostically important. Items were derived based on literature review and dementia expert input to represent a diverse array of observable behaviors known to occur during one-on-one interactions with these patients in a medical setting. Thus, the measure was not intended to detect only a single syndrome (e.g., bvFTD), but to yield elevations on at least some items regardless of which dementia syndrome is present. This study examined SBOCL results in well-characterized patients with bvFTD, svPPA, nfvPPA, AD, and PSP syndromes and found significant behavior symptom elevations in all five groups, with partially overlapping but divergent patterns of score elevations across syndromes. Predictably, bvFTD patients scored significantly higher than patients in other groups on the majority of items, though svPPA patients showed significantly worse anxiety than patients with other syndromes. There was substantial behavior overlap across syndromes, however, and thus the differential diagnostic utility of the SBOCL for pinpointing a single disorder may be more modest, particularly if used in isolation without additional clinical information or testing. Thus, it appears the measure is best for detecting “dementia-specific behaviors” that identify patients who are likely not neurocognitively normal, with some secondary usefulness in providing differential diagnosis between syndromes.

This study showed that the SBOCL demonstrates a number of useful psychometric characteristics for its intended purpose. First, preliminary scores for interrater reliability in a subset of the sample were strong, with “moderate,” “good,” and “very good” reliability across items for video raters, and only slightly reduced reliability between an in-room rater and two video raters. It was notable that the item with poorest interrater reliability included medical jargon that may have been difficult for untrained examiners to understand (“Was stimulus-bound”). Another psychometric strength of the measure was its level of accuracy in discriminating patients with each of the neurodegenerative syndromes from older healthy controls. For this purpose, it appears that the frequency-by-intensity product score was a more useful measure than whether the behavior was present or absent (Table 2). bvFTD patients were best differentiated from controls because they failed to adapt to the structure of testing and were stimulus-bound. Patients with svPPA were more likely than controls to be overly dependent and self-conscious, and failed to adapt to the testing structure. Items best differentiating AD patients from controls were their tendency to be self-conscious and their fluctuating levels of cognitive ability, and patients with nfvPPA were more likely to be self-conscious than controls. AUC's for the Disorganized score, using a cutoff of ≥2, were above 90% for bvFTD and PSP syndromes, over 82% for svPPA and AD syndromes, and 76% for nfvPPA patients, and positive predictive value in almost all groups was 100%, with false omission rates at 21% or less. This indicates that no healthy controls scored at or above 2 on the Disorganized score but that the majority of patients with dementia syndromes did, highlighting the usefulness of this score as a screening measure to help a non-specialist identify which patients to refer for further evaluation. The Reactive score was most accurate with a cutoff score of 3, and was most sensitive with the svPPA group (AUC vs. controls was 88%). This score also showed moderate sensitivity discriminating AD and nfvPPA patients from controls (AUC: 77 and 73%), while this score was less useful for PSP and bvFTD patients. PPV was 100% for all groups on this measure, meaning that no healthy control scored at or above the cutoff. Finally, the Insensitive score performed more weakly than the other two scores, with greatest patient discrimination in the bvFTD group (AUC = 74%), but with fairly poor discrimination in the other syndromes (AUCs ranging from 64 to 52%), and with fairly high false omission rates.

The brain-behavior analyses of the three subscale scores provide additional psychometric validation by supporting the construct validity of the SBOCL. The regions showing a linear relationship between both Disorganized and Reactive score spontaneous behaviors and gray matter volume loss were primarily areas throughout the frontal and temporal lobes that have been strongly associated with behavior (36–38). These measures can predict volume loss predominantly in these socioemotional brain regions, rather than other regions related to memory, language, or visuospatial skills, and the prediction holds regardless of diagnostic group membership, which suggests that the construct these SBOCL subscale scores is measuring is indeed behavioral. The brain-behavior correspondence of the Insensitive subscale appears to reflect the score elevations seen only within the bvFTD group, and thus the construct validity of this score should be considered weaker. Brain-behavior correspondence of individual items within the cluster may have varying strengths and divergent regional specificity.

Overall, the SBOCL has a number of features that lend it potential utility in either clinical research or possibly clinical settings. Because it is an observer-based measure the SBOCL can be completed by a clinical observer in <2 min, after the visit with the patient is concluded, which makes it appropriate for settings where only limited time is available with the patient. The gold standard for evaluation of dementia patient behavior remains an interview of a knowledgeable informant; however, this measure many be an alternative when no informant is available. The SBOCL performed with solid psychometric properties in this study despite being derived from behavioral observations made by student research assistants, suggesting that the SBOCL has utility in settings where a non-expert user wishes to detect the behavioral features of dementia. Because of these features, it may be particularly useful for screening, and on the basis of further validation, might have the potential to be used in a clinical setting could yield information that would suggest referral to a dementia specialist for further evaluation. In a research setting, this measure might be used to quickly ascertain the clinical profile and behavioral severity of a patient being screened for enrollment in a study that will perform more detailed testing to determine their specific neurodegenerative syndrome.

An important consideration for this preliminary validation study was that it was performed in a clinical research setting, and with particular parameters (e.g., after 1 h of one-on-one interaction with the patient which included cognitive testing); thus, more precise estimates of the utility of the SBOCL in any specific clinical or research setting with alternate parameters must still be demonstrated through additional investigation. In particular, it remains unclear whether it would be useful for shorter patient encounters, during which there would be fewer opportunities for these behaviors to be observed. It also remains to be determined whether these behaviors would be as easily elicited during a standard clinical interview or medical evaluation, either in a research or clinical setting, or while other family members are present. The individuals completing the SBOCL for this study were typically research assistants with an undergraduate level of education and thus with no formal clinical training; however, it could also be argued that because they were employed at a clinical research center specializing in neurodegenerative disease, they might have some expertise in dementia behavior that could have affected their responses. Also, while this study primarily enrolled patients at the very mild/mild stage of disease progression, patients were already demented, and were not further subdivided by disease severity, thus additional study is needed to characterize the sensitivity of the SBOCL at the earliest symptomatic (i.e., mild cognitive impairment, or MCI) stages of these syndromes. To improve its differential diagnostic utility, additional studies should also be performed to determine how the SBOCL profiles of dementia patients differ from the profiles of individuals with psychiatric disorders, or whether these profiles are significantly influenced by clinical confounds such as substance abuse, medications, or sleep disturbance. This sample was in general highly educated, and additional studies determining the influence of educational and other sociocultural factors would be of value. However, these preliminary validation data suggest that the SBOCL is a useful measure that allows individuals without particular medical training or dementia expertise to identify behavioral features consistent with a number of dementia syndromes, without adding time to a patient encounter or requiring the presence of an informant, and thus has the potential to improve dementia screening in a variety of settings.

## Data Availability Statement

The raw data supporting the conclusions of this article will be made available by the authors, without undue reservation.

## Ethics Statement

The studies involving human participants were reviewed and approved by University of California San Francisco Institutional Review Board. The patients/participants provided their written informed consent to participate in this study.

## Author Contributions

KR, GT, LG, TW, and TS-U contributed to the conception and design of the study. KR, JK, and BM provided participants for study. KR, GT, and LG performed data analysis. GT and RL performed brain imaging analysis. KR, GT, LG, and TW wrote sections of the first draft of the manuscript. PC and MK performed data management and contributed substantial edits and revisions to the first draft. All authors contributed to editorial revision of the final draft and approved the submitted manuscript.

## Funding

Funding for this work was provided by the NIH National Institute on Aging (R01 AG029577, PPG P01 AG1972403, and P50 AG023501), and the Larry L. Hillblom Foundation (2014-A-004-NET).

## Conflict of Interest

The authors declare that the research was conducted in the absence of any commercial or financial relationships that could be construed as a potential conflict of interest.

## Publisher's Note

All claims expressed in this article are solely those of the authors and do not necessarily represent those of their affiliated organizations, or those of the publisher, the editors and the reviewers. Any product that may be evaluated in this article, or claim that may be made by its manufacturer, is not guaranteed or endorsed by the publisher.
